# Exploring the Factors Influencing Consumers to Voluntarily Reward Free Health Service Contributors in Online Health Communities: Empirical Study

**DOI:** 10.2196/16526

**Published:** 2020-04-14

**Authors:** Junjie Zhou, Fang Liu, Tingting Zhou

**Affiliations:** 1 Shantou University Business School Shantou China; 2 China Life Property & Casualty Insurance Company Limited Beijing China; 3 Henan Foreign Trade School Zhengzhou China

**Keywords:** telemedicine, health services, social media, reward, social interaction, social support, pay-what-you-want

## Abstract

**Background:**

Rewarding health knowledge and health service contributors with money is one possible approach for the sustainable provision of health knowledge and health services in online health communities (OHCs); however, the reasons why consumers voluntarily reward free health knowledge and health service contributors are still underinvestigated.

**Objective:**

This study aimed to address the abovementioned gap by exploring the factors influencing consumers’ voluntary rewarding behaviors (VRBs) toward contributors of free health services in OHCs.

**Methods:**

On the basis of prior studies and the cognitive-experiential self-theory (CEST), we incorporated two health service content–related variables (ie, informational support and emotional support) and two interpersonal factors (ie, social norm compliance and social interaction) and built a proposed model. We crawled a dataset from a Chinese OHC for mental health, coded it, extracted nine variables, and tested the model with a negative binomial model.

**Results:**

The data sample included 2148 health-related questions and 12,133 answers. The empirical results indicated that the effects of informational support (β=.168; *P*<.001), emotional support (β=.463; *P*<.001), social norm compliance (β=.510; *P*<.001), and social interaction (β=.281; *P*<.001) were significant. The moderating effects of social interaction on informational support (β=.032; *P*=.02) and emotional support (β=−.086; *P*<.001) were significant. The moderating effect of social interaction on social norm compliance (β=.014; *P*=.38) was insignificant.

**Conclusions:**

Informational support, emotional support, social norm compliance, and social interaction positively influence consumers to voluntarily reward free online health service contributors. Social interaction enhances the effect of informational support but weakens the effect of emotional support. This study contributes to the literature on knowledge sharing in OHCs by exploring the factors influencing consumers’ VRBs toward free online health service contributors and contributes to the CEST literature by verifying that the effects of experiential and rational systems on individual behaviors can vary while external factors change.

## Introduction

### Background

With the development of information and communication technologies (ICTs), the sharing economy (SE) has emerged as a market for collaborative consumption in which peer communities gain access to a pool of shared knowledge and resources [1-3]. Health services, a typical kind of knowledge-intensive service [4,5], has recently become increasingly popular worldwide on many noncommercial web-based SE platforms. Such services emerge in online health communities (OHCs)—a special kind of online forums that links health care professionals and normal users [6-10]. In OHCs, health care professionals and consumers collaborate with each other to generate new health knowledge, such as disease symptoms and routine daily care discussions, health self-management experiences, or suggestions on treatments [5,11-19]. The generated knowledge will become available to the public and can be freely accessed by every consumer on online SE platforms [20,21].

Similar to many other noncommercial web-based SE platforms, OHCs are facing the sustainability issue (ie, the provision of free health knowledge and health services) [6,22-25]. In OHCs, health care professionals or enthusiastic consumers generally provide free health knowledge and health services. They voluntarily contribute their time and knowledge to the community [11,22,26]. However, both health care professionals and other free health service contributors have their own professional burnouts, duties, and responsibilities [22,27,28]. They are likely to stop providing health knowledge and health services if they lose their passion to contribute or they become busy with other duties.

### Objectives

To keep the sustainable provision and sharing of free health knowledge and health services, some OHCs have designed a new feature that allows consumers to voluntarily reward free health service contributors. Such rewarding behavior is particularly important for OHCs to thrive, because the rewards act as monetary incentives that can stimulate health service providers to continuously contribute high-quality health knowledge and free health services [28-35]. However, given that the voluntary reward feature is new and consumers’ rewarding behaviors are emerging, we still have little knowledge on the following questions:

1. What are the factors that motivate consumers to voluntarily reward free health service contributors in OHCs?
2. How do those factors motivate consumers to voluntarily reward free health service contributors in OHCs?

This study aimed to address the abovementioned questions. We adopted the cognitive-experiential self-theory (CEST) as the theoretical foundation and proposed seven hypotheses. We crawled an objective dataset from an OHC for mental health and verified most of the hypotheses. The empirical results indicate that informational support, emotional support, social norm compliance, and social interaction positively influence consumers to voluntarily reward free online health service contributors. Social interaction enhances the effect of informational support but weakens the effect of emotional support. These findings provided several important theoretical contributions and practical implications.

## Methods

### Literature Review

We reviewed two streams of related studies to address the research questions. Specifically, we reviewed the literature on free health services in OHCs to describe the characteristics of free online health services. We reviewed the literature on pay-what-you-want to understand the theories, variables, and models that were used to explain consumers’ voluntary rewarding behaviors (VRBs). In this section, we have summarized the implications of prior studies.

#### Free Health Services in Online Health Communities

There are different types of OHCs (eg, peer communication for health care professionals, physician-patient interaction communities, and patient-patient interaction communities), and activities in different OHCs are organized differently [7,11,30,36]. In this study, we have specifically focused on freemium problem-solving communities (eg, health-related question and answer forums), in which both health care professionals and patients can participate [17,22,36,37]. In those communities, health services—eg, users’ collaborative behaviors to generate new health knowledge, help consumers meet their health needs, and help consumers to reach a better state of health [8,38-41]—are usually free, and both health service providers and consumers create new values in a collaborative way [9,15,21,38]. As a voluntary behavior, providing health services is mainly motivated by prosocial factors. Prosocial factors are those factors relating to a broad range of actions intended to benefit one or more people other than oneself, such as trust, enjoyment, altruism, empathy, and reciprocity [9,11], and such factors are usually salient in noncommercial web-based communities [2,42]. Such factors are important because they enable the sustainable provision of free health services in OHCs [11,22,24].

Free web-based health services provide consumers many benefits. Consumers can conduct health-related activities in OHCs, such as health knowledge sharing and seeking (eg, recommending treatment plans and seeking health care suggestions) and health self-management [5-7,12,36,43]. They can manage their embarrassing conditions or stigmatized illnesses in OHCs and access health services without physically appearing in hospitals [22,36]. Free online health services meet consumers’ needs and help them to achieve better health outcomes, such as higher electronic health literacy, increased patient empowerment, and a better quality of life [6,8,39,44-46].

The nature of free health services in OHCs can be treated as social support [6-8,19,36,47,48]. Social support refers to the individual’s perceptions and experiences that they feel they are being cared for [49]. Social support could be divided into five subtypes: informational support, emotional support, network support, esteem support, and tangible support [49]. In this paper, we have particularly focused on freemium problem-solving communities, and in such communities, consumers mainly exchange emotional support (eg, show or receive sympathy and make new friends or companionships) and informational support (eg, health knowledge seeking or contributing), whereas network support, esteem support, and tangible support are less salient. For example, many studies have shown that members in such communities do not form new subnetworks [16,36,47]. As a result, informational support and emotional support become the two most crucial aspects in the literature relating to freemium problem-solving communities [8,9,36,47,50-52]. In this paper, we have followed prior studies and adopted informational support and emotional support to describe the contents of free online health services.

#### Pay-What-You-Want and Voluntary Rewarding

Voluntarily rewarding free health services belongs to an emerging business model that gives consumers full control in monetizing free web-based knowledge/goods/services [33,34]. Consumers can choose to pay any amount of money or pay nothing but still enjoy free knowledge/services. Such behavior is similar to the pay-what-you-want behavior, which is “a new participative pricing mechanism in which consumers have maximum control over the price they pay” [3]. According to existing literature, firms can use pay-what-you-want pricing for two different goals: (1) commercial profit or (2) free promotion to increase knowledge and service provision on the internet [53]. In this paper, we believe that voluntary behavior is noncommercial behavior that is similar to the pay-what-you-want behaviors for the promotion of increasing knowledge/service on the internet. In such a situation, exchange partners build their relationships according to prosocial exchange norms (eg, norms of reciprocity, norms of cooperation, or norms of distribution) [54,55]. Thus, we have referred to the studies on pay-what-you-want behaviors and investigated consumers’ voluntary reward behaviors from a prosocial motivation perspective [2,3,54,56,57]. We reviewed some related studies and summarized them in Table 1.

**Table 1:** Key constructs related to the pay-what-you-want behaviors in prior studies.

Sources	Contexts	Theory	Independent variables	Dependent variables
Kim et al [3]	Restaurant, cinema, and delicatessen	Equity theory	Fairness, altruism, satisfaction, and loyalty CVs^a^: price consciousness and income	Final price paid
Jang and Chu [58]	Experiments for consumers	Equity theory	Fairness motives of individuals, self-signaling, and norm conformity	Willing to pay
León et al [59]	Travel company	Game theory	Customer characteristics, the influence of subjective factors, and product characteristics	Payments in El trato
Hilbert and Suessmair [60]	A laboratory experiment about a travel mug	N/A^b^	Social interaction and social norm compliance	Willing to pay
Regner [57]	An online survey about the online music label/store, Magnatune	N/A	Social preferences, reciprocity, guilt, social norms, altruism, fairness, and social image concerns	Willing to pay
Barone et al [61]	A leadership questionnaire	N/A	Consumer power, perceived value, and perceived self-reliance	Purchase intentions
Dorn and Suessmair [62]	Survey in several countries under three hypothetical situations where a McDonald’s Big Mac was offered	N/A	Satisfaction, income, price consciousness, reference price, high level of reputation, loyalty, altruism, fairness, social acceptance, and social norm compliance	Willing to pay
Narwal and Nayak [63]	Scenario-based online experimental approach on purchase intention	N/A	Quality of product/services, satisfaction, types of products/services, self-image, and fairness perception Moderators: communication message, interaction, and reference prices	Pay-what-you-want
Viglia et al [64]	Service	Fairness theory	Timing and uncertainty reduction	Consumers’ chosen payments

^a^CV: control variable.

^b^Not applicable.

#### Implications of Prior Literature for This Study

We concluded three useful findings according to the literature review. First, pay-what-you-want is a result of consumers’ positive experiences with the services via direct interactions with service providers [59,65]. The experiences are related to factors in three domains: (1) consumer characteristics, eg, fairness motivation, income, or self-image [3,57,62], (2) product or service content–related factors, eg, price, quality, or value of services [59,61,66], and (3) interpersonal factors, eg, social interaction or social norm compliance [60,62,66].

Specific to this study, we proposed that consumers’ VRBs toward free health service contributors are a result of consumers’ positive experiences with the services via direct interactions with service providers in OHCs [59,65]. In addition, we incorporated informational support and emotional support as service content–related factors, social norm compliance as interpersonal factors, and social interaction to describe the communication between service providers and consumers [60,66].

Second, research focuses are shifting with time. As discussed above (please see the timeline of prior studies in Table 1 and the first paragraph of section: Implications of Prior Literature for This Study), early studies have adopted the experimental approach and mainly focus on consumer characteristics, whereas recent studies have paid more attention to service content–related factors and interpersonal factors (see also Table 1). For example, scholars have verified that the ways in which online health services are delivered are crucial in the era of ICTs [39,67], and consumers can easily be influenced by peers or friends their age [62,63]. In addition, new methodologies, such as online surveys [57,62] and econometric modeling based on objective data, are emerging [22,30]. We sought to adopt new methodologies in this paper.

Finally, there is a lack of conceptual frameworks in analyzing consumers’ pay-what-you-want behaviors. Scholars tend to analyze this issue from a prosocial motivation perspective. They have adopted theories such as the equity theory and fairness theory to select influencing factors (see Table 1) rather than using them to build proposed research models. Scholars should build a conceptual framework to better explain consumers’ pay-what-you-want behaviors [68].

### Theoretical Foundations and Logic for Model Development

#### Cognitive-Experiential Self-Theory

As there is a lack of conceptual frameworks to explain consumers’ pay-what-you-want behaviors [68], we incorporated a new theory, the CEST, to explore how the four selected factors influence consumers’ VRBs in OHCs.

CEST is a psychological theory that argues that human beings operate with two systems: an experiential/intuitive system (hereafter referred to as the experiential system) and a rational/analytical system (hereafter referred to as the rational system) [69-71]. We noted in persuasion literature that scholars also refer to the dual-process models (ie, the elaboration-likelihood model and the heuristic-systematic model) [72,73]. These models also mentioned controlled vs automatic processes. However, these models are limited to validity-seeking persuasion contexts [73], which are not suitable in our research contexts. Specifically, they assume that the primary goal of recipients is to assess the validity of persuasive messages [73], but in our research contexts, the rewarding behaviors are voluntary, and people post the answers and discussions in OHCs to help rather than to persuade users to reward. Compared with assessing the validity, assessing the helpfulness is more important for recipients. The experiential system operates in an automatic, nonverbal, imagistic, rapid, and effortless manner, which is associated with affect or emotions. This system has also been called an automatic system [74], a natural system [75], and system 1 [76]. Compared with the experiential system, the rational system is a reasoning system that operates in a conscious, verbal, abstract, slow, and effortful manner, which is affect-free and demanding of cognitive resources [70,71]. This system has also been called an intentional system [74], an extensional system [75], and system 2 [76].

CEST is being widely used to explain consumers’ web-based behaviors, including their web purchase–related decisions. For example, consumers’ reactions to experiential information demonstrates a contagion effect: experiential information at the early stage can cause more similar information in the following stage, and normal consumers like to follow opinion leaders who post experiential information [77]. To avoid consumers being influenced by negative experiential information, operation teams should enhance the information or topic management in their communities [78]. In their study, Kim and Lennon [79] applied CEST to explain the effects of different product presentation formats (visual vs verbal) on consumers’ attitudes toward products and their purchase intentions in an electronic-commerce context. Previous research has verified that consumers’ involvement and their consequential behaviors (eg, attitude and purchase attention) are conditional upon the amount of experiential information provided by web-based sellers [80]. The abovementioned studies indicate that consumers’ money-related decisions could be explained with CEST. Therefore, it is appropriate to use CEST to explain consumers’ VRBs in OHCs.

#### Key Logic for Model Development

We built our research model based on the following logic.

According to CEST, the rational is a verbal reasoning system—it suggests that human behaviors are driven by logic inferences from the information or evidence received [70]. As discussed earlier, informational support is one of the most important aspects of free health services in OHCs. Consumers evaluate the quality of health services they receive (eg, whether the services include useful health knowledge) and then decide how to react to these services (eg, whether to reward or not). This is a reasonable and logic-directed process. We thus used the impact of informational support to reflect the rational processing [70].

According to CEST, the experiential system is an affect-driven system—it suggests human behaviors are directed by pursuing positive feelings and avoiding negative feelings [70]. On one hand, emotional support is closely related to affect, because emotional support is a typical feeling of experience and intuition [36]. As a result, we used the impact of emotional support to reflect the experiential processing. On the other hand, consumers can observe what others do and comply with others to avoid negative results [57,62]. We thus used the impact of social norm compliance to reflect the experiential processing.

**Figure 1:**
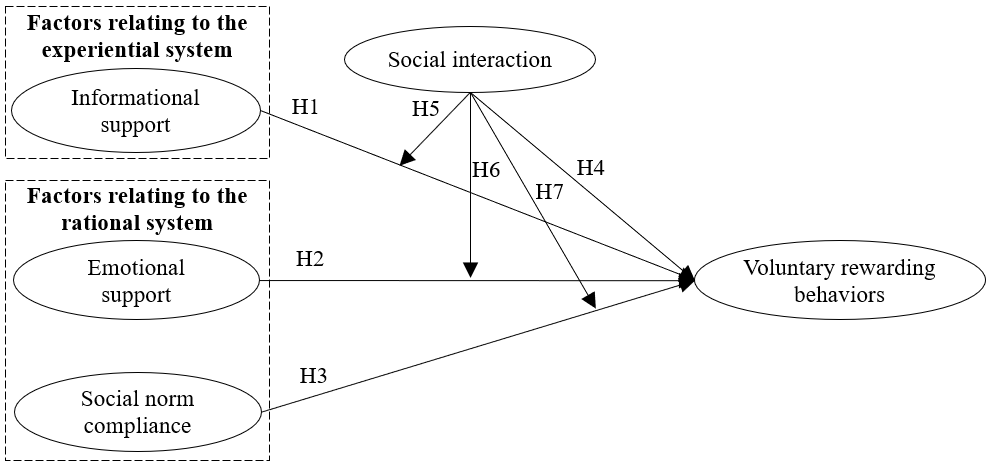
Hypotheses and research model.

CEST also argues that the relative influence of both systems varies along a dimension of complete dominance by one system to complete dominance by the other [70]. Previous studies have verified that external factors could change the effects of experiential and rational systems [81,82], which is followed in this study. Considering that consumers’ experience of health services is influenced by the interaction between service providers and consumers (ie, social interaction) [5,39,83], we treated social interaction as an external factor and proposed that social interaction can change the effects of emotional support, social norm compliance, and informational support (see Figure 1).

### Hypotheses

#### Direct Effects Relating to the Rational System

Informational support refers to the overall quality and usefulness of the information received in OHCs. According to CEST, the rational system is verbal and based on the information received, so users tend to rely on rational processing when receiving informational support. Service providers and consumers usually collaboratively generate health services in the form of question and answers in OHCs. Consumers post their questions and respondents address these questions. They discuss health-related issues and generate new health knowledge in OHCs. CEST also suggests that by rational processing, consumers behave based on the logical inference from information/evidence received [70]. As a result, to better help consumers achieve logical inference, the information or knowledge quality provided in OHCs becomes important. High quality usually causes positive results. For example, high-quality information can satisfy consumers’ informational needs [84,85] and motivates users to purchase [86] or to continue using web-based services [87]. Specific to the context of health services, consumers will evaluate the quality of health information they receive from free health services. As CEST suggests, if the consumers’ rational processing of information suggests that it is logical and can meet their health-related needs, they will be more likely to reward these services [70]. Thus, we hypothesized the following:

H1: Informational support expressed in free health service threads positively influences consumers’ voluntary rewarding behaviors in OHCs.

#### Direct Effects Relating to the Experiential System

Emotional support refers to sympathy, ie, perceiving, understanding, and reacting to others’ distress or needs [88]. According to CEST, the experiential system is emotional [70], so users tend to rely on experiential processing when receiving emotional support. As the experiential system suggests that users are motivated to pursue positive emotions and avoid negative emotions [70] when receiving emotional support, consumers’ consequential behaviors (eg, rewarding decisions) are directed by their experiential processing [70]. Specifically, consumers participate in OHCs to look for patients similar to them. They can share personal experiences and exchange emotional support. Expressing care and concern could make others feel that they are being taken care of and are valued [36]. Emotional support is especially important for consumers with diseases who rely less on physical treatments. For example, consumers with mental health conditions can be alleviated with emotional direction and confession and can move to a better state of health [22,36]. Consumers in turn are likely to reward these services that provide them useful emotional support. Thus, we hypothesized the following:

H2: Emotional support expressed in free health service threads positively influences consumers’ voluntary rewarding behaviors in OHCs.

Social norm compliance refers to conformity to a set of norms that are accepted by a significant number of people in a social surrounding, community, or society [60,62]. The detailed norms in prior studies include altruism, reciprocity, and fairness [3,57,60]. According to CEST, the experiential system learns from prior experience, belief, or norms [70], so consumers tend to rely on experiential processing when they feel they need to comply with some social norms. Normative messages can influence people and promote prosocial behaviors [89,90]. In a free service and voluntary reward context, service providers help others without expecting economic rewards. This is a prosocial behavior and can activate the service consumers’ sense of reciprocity and fairness. CEST also suggests that the experiential system influences consumers to pursue positive emotions and avoid negative emotions [70]; therefore, we believed that social norm compliance can positively influence consumers to conduct voluntary reward behaviors to pursue positive feelings and avoid negative feelings [57,60,62,70]. Thus, we hypothesized the following:

H3: Social norm compliance positively influences consumers to voluntarily reward free health service contributors in OHCs.

#### The Direct Effect of Social Interaction

Social interaction refers to the observed strength of relationships, the amount of time spent, or the communication frequency among health service providers and consumers in a health service thread [39,91,92]. The application of ICTs in health care has significantly changed the context in which health service is delivered and experienced [5,83]. Consumers need to interact with service providers to better understand professional health knowledge and know how to apply it [39]. More frequent social interactions between service providers and consumers can better deliver health services and make consumers have better health outcomes [93,94]. Consumers could be grateful to service providers and thus choose to reward those free health services to feel less guilty [60,68]. Namely, social interaction drives consumers to pursue positive feelings and avoid negative feelings [70]. Thus, we hypothesized the following:

H4: Social interaction between service providers and consumers motivates consumers to voluntarily reward online free health service contributors in OHCs.

#### Moderating Effects of Social Interaction

CEST suggests that the extent to which people think or behave primarily according to the experiential system or rational system depends on the situation [70]. The relative influence of both systems varies along a dimension of complete dominance by one system to complete dominance by the other [70,95]. Previous studies have verified that external factors could change the effects of experiential and rational systems [81,82]. For example, in a conflict‐handling context, constructive thinking together with the experiential system and rational system influences consumers’ conflict‐handling style [81]. In an online shopping context, consumers’ involvement changes the effects of experiential information on their product attitude and purchase intention [80]. We followed the above findings and proposed that social interaction as an external factor changes the effects of the experiential system and rational system on consumers’ VRBs.

OHCs are web-based social networks in which health-related stakeholders with common interests, goals, or practices interact to share health information and knowledge, communicate health services, and engage in social interaction [7,91]. It is the nature of social interaction and the resources embedded within social interaction networks that sustain the communities [91]. In OHCs, social interaction links different community members and provides them opportunities to discuss health information and knowledge [93,96]. We proposed that higher levels of social interaction can facilitate consumers to think or behave in a manner that is based more on the rational system. This is because by interacting with others, consumers can clearly express their health condition and needs, which also helps knowledge providers to better understand their needs and thus offer more useful suggestions. Consumers can then also carefully compare different information they receive. During the above mentioned process, they take time to think and logically evaluate the quality of informational support, which also slows down their decision-making process. Given that the rational system is slow and more logical, consumers’ VRBs rely more on their rational system [70,95], meaning they rely more on informational support. Thus, we proposed the following:

H5: Social interaction positively moderates the effect of informational support on consumers’ voluntary rewarding behaviors in OHCs.

As discussed earlier, both emotional support and social norm compliance are factors relating to the experiential system. According to CEST, because the relative influence of the experiential system and rational system varies from complete dominance by one to complete dominance by the other [70], when consumers rely more on their rational system to decide whether or not to reward, they tend to rely less on their experiential system, ie, although higher levels of social interaction make consumers rely more on informational support, it also makes users rely less heavily on emotional support and social norm compliance. In addition, when consumers are highly involved in social interaction, they pay more attention to the informational support they receive; therefore, they tend to be less influenced by their emotions and social norms [91]. Thus, we proposed the following:

H6: Social interaction negatively moderates the effect of emotional support on consumers’ voluntary rewarding behaviors in OHCs.

H7: Social interaction negatively moderates the effect of social norm compliance on consumers’ voluntary rewarding behaviors in OHCs.

### Data Collection

To test the hypothesized model, we crawled an objective dataset from the question and answer forum on a Chinese OHC for mental health (the question and answer forum on YiXinLi). YiXinLi is a leading web-based health community for mental health in China. We focused on mental health because without mental health there can be no true physical health [97]; in addition, mental health services in China are relatively limited [98,99], and consumers usually read books or use the internet for health-related knowledge or services [100].

YiXinLi was set up in 2011 and aims to promote mental health services in China. The question and answer forum on YiXinLi, which was launched in 2014, provides free mental health services for consumers. Consumers can post their health-related questions in the question and answer system and wait for free answers. However, with the emerging trend of knowledge monetizing [33,34], the YiXinLi website launched a new feature, “Voluntary Reward,” that supports the consumers’ decision to reward the answers as they desire. As rewarding is voluntary, we were curious about the factors motivating consumers to voluntarily reward free health services and the impact of those factors.

**Figure 2:**
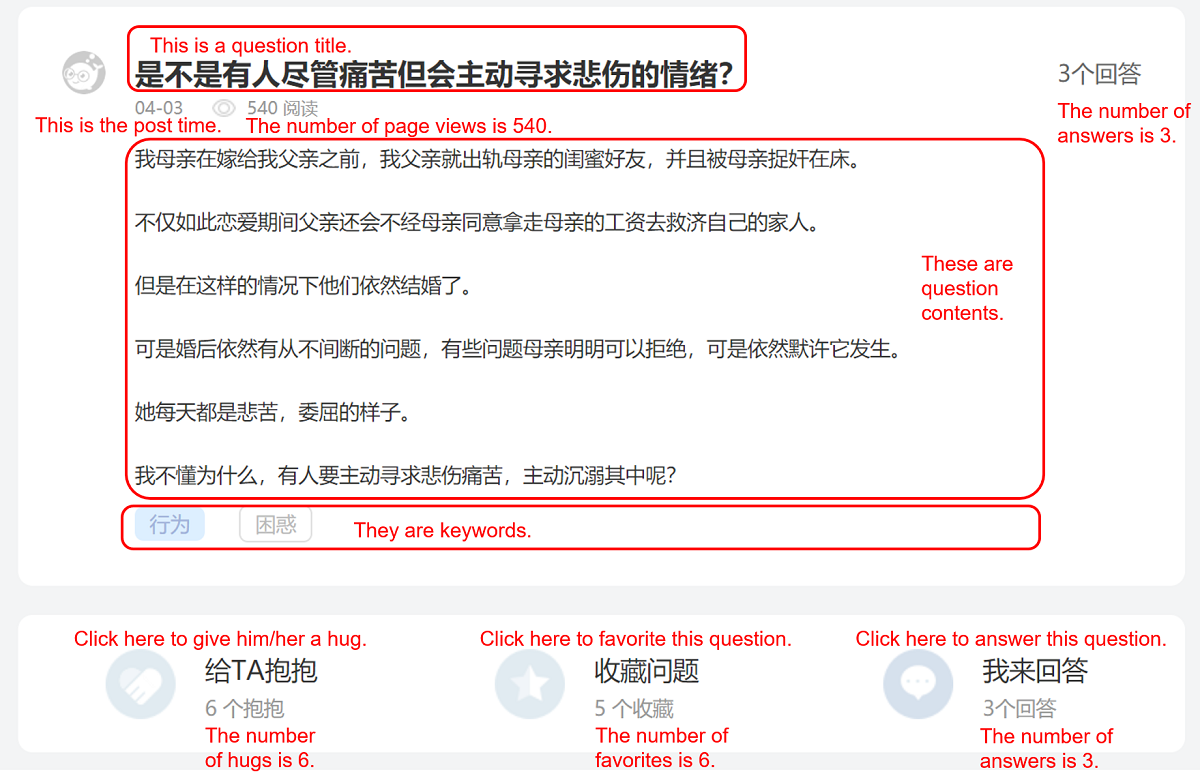
A sample of a question.

We used a spider program (named Locoy Spide) and crawled all the threads on the YiXinLi question and answer forum on January 12, 2019. We treated a question and answer thread (ie, a question and its answers) as the basic analysis unit. We cleaned the data by deleting 12 inconsistent threads—the threads in which the actual number of answers was less than the number shown on the web page because one or more answers were deleted by the providers (the number of answers displayed on the web page includes all the answers that have been provided. However, if a provider deletes his or her answer, the number shown on the web page does not change, but the actual number of answers we crawled would be less than the number shown on the web page). After cleaning the data, we had 2148 data samples, including 2148 questions and 12,133 answers. Figure 2 shows detailed information on a question posted in a question and answer thread.

**Figure 3:**
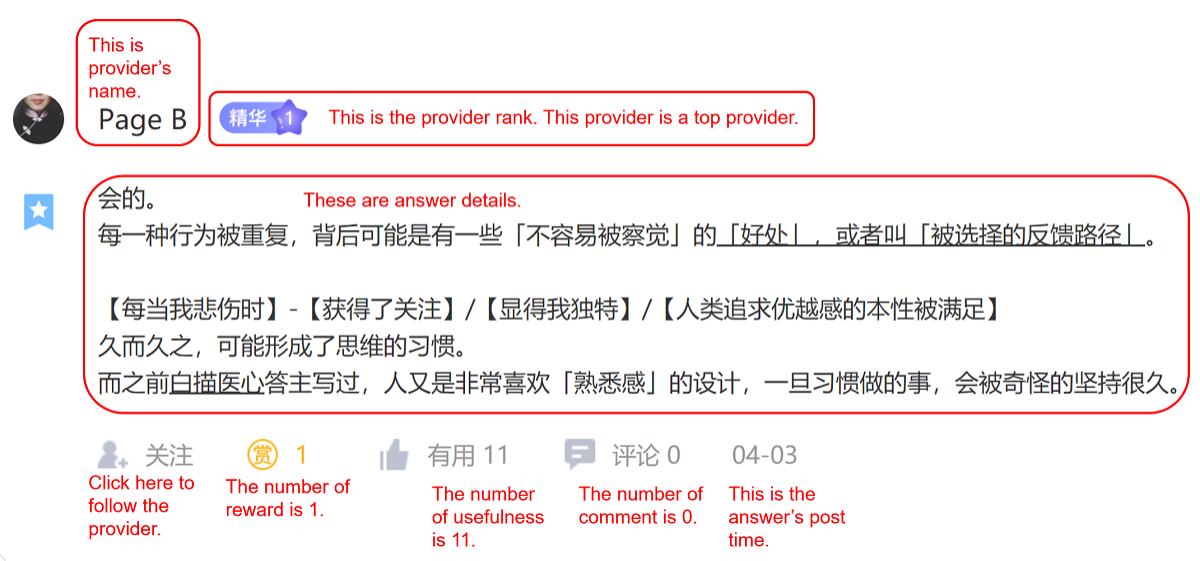
A sample of an answer.

As shown in Figure 2, the question and answer thread web page displays question-related information (eg, question title, question content, post time, number of page views, number of answers received, number of hugs received, and number of times favorited) at the top of the page. Figure 3 shows detailed information on answers in a question and answer thread.

As shown in Figure 3, the question and answer thread web page displays answer-related information (eg, provider ID, provider rank, answer details, post time, number of rewards, usefulness rank, and number of comments) following the question.

### Data Coding

We coded nine variables that were used for data analysis. We treated consumers’ *voluntary rewarding behaviors* as the *dependent variable*. *Voluntary rewarding behaviors* was measured by the number of times a thread is rewarded. There were four key *independent variables*: *informational support*, *emotional support*, *social norm compliance,* and *social* *interaction*. Other factors such as *answer length* [101], *date of exposure*, *page view* [102], and *provider reputation* [103] also might influence consumers’ rewarding behaviors and were treated as control variables in this study. Table 2 shows the details of all variables.

The descriptive statistical results of different variables are shown in Table 3.

**Table 2:** Variables and measurement.

Variable	Value, mean (SD)	Measurement
VRB^a^	2.141 (3.334)	The VRB is measured by the rewarding times of a thread received. For example, the answers of the sample thread in Figure 3 received four rewards (3+1+0=4). Therefore, the value of VRB is 4 We did not use the sum of real money that all answers received. In fact, we cannot capture the actual sum of rewarded money in a thread
IS^b^	4.375 (3.991)	On YiXinLi, consumers can evaluate the answer quality with the feature, *usefulness*. We measured IS with the average answer quality in a thread For example, the answer in Figure 3 have received 25 times of usefulness. And if there is another answer for the same question received 14 times of usefulness; in total, the question and answer thread received 25 times of usefulness. The value of IS is assigned as 8.333 (ie, 25/3=8.333)
ES^c^	3.274 (1.467)	On YiXinLi, providers and other consumers can use the feature, *hugs*, to show their sympathy for help-seekers We thus use the volume of *hugs* in a thread to measure the emotional support that help-seekers received. For example, there are six hugs in Figure 2. Thus, the value of emotional support is 6 Although *hugs* in a thread are expressed to the help-seeker (ie, the thread poster), the empathy mechanism (ie, feeling there are patients like me) makes other consumers who have similar conditions feel that they are being taken care of and loved by others
SNC^d^	0.536 (0.61)	SNC is measured by the percentage of people interested in the question who finally reward the question. Such a measurement reflects the peer pressure the consumers feel when they find that others have rewarded the thread they viewed. We designed this measurement according to industrial practice and prior studies. Previous literature suggests that other consumers’ purchase behavior (number of goods purchased) acting as social norms influences a focal consumer’s intention [104]. For example, in the electronic commerce context, Amazon designed a notification stating “15% of consumers who viewed this item have bought this item” to incent other consumers’ purchase intention/behaviors; in the tax auditing context, some scholars used the rate of taxpaying (tax paid/tax owed) to measure the compliance rate (ie, other people’s paying behaviors) and verified that individuals’ taxpaying intention will increase when they can see a higher compliance rate [105]. We followed the above studies and measured SNC with the following equation: *SNC=VRB/(favorite+1)* Specifically, VRB refers to the number of rewarding. The volume of *favorite* (see Figure 2) represents the number of consumers who are interested in a question. “1” represents the help-seeker himself/herself, and *(favorite+1)* represents all the people who are interested in a question. The result of *VRB/(favorite+1)* therefore represents the compliance rate (ie, the percentage of people interested in the question who finally reward the question) For example, there are five favorites in Figure 2. Thus, the value of SNC is 0.83 (ie, 5/(5+1)=0.83)
SI^e^	8.75 (8.757)	SI is measured by the interaction frequency between service providers and consumers in a thread. On YiXinLi, providers can respond to a question by posting their answers. Providers and consumers can also discuss a particular answer via the feature *comment* (see Figure 3). Social interaction is evaluated by the sum of answer volume and comment volume For example, there are three answers and 0 comments in Figure 3. Thus, the value of SI is 3 (ie, 3+0=3)
AL^f^	188.4 (120.866)	AL refers to the average text length of all answers in a thread. We calculated the character numbers of all answers and then divided the volume of answers in a thread For example, there are three answers in a thread. The first one has 200 characters, the second one has 300 characters, and the last one has 400 characters. Thus, the value of AL is 300 (ie, (200+300+400)/3=300)
DoE^g^	73.17 (135.115)	DoE is measured by comparing the time a question is posted with the time we crawled the dataset
PV^h^	647.985 (1918.211)	PV refers to how many times a thread is read. For example, the thread in Figure 2 was read 171 times. Thus, the value of *PV* is 171.
PR^i^	0.835 (0.193)	On YiXinLi, there are 3 rank levels for a service provider, ie, normal provider, higher-rank provider, and top provider. The rank level is related to how many times their answers were set as best answers. We used the rate of higher rank/top providers of all providers in a thread to measure the PR For example, the three providers in a thread include one normal provider, one higher-rank provider, and one top provider. Thus, the value of PR is 0.667 (ie, 2/3=0.667).

^a^VRB: voluntary rewarding behavior.

^b^IS: informational support.

^c^ES: emotional support.

^d^SNC: social norm compliance.

^e^SI: social interaction.

^f^AL: answer length.

^g^DoE: date of exposure.

^h^PV: page view.

^i^PR: provider reputation.

**Table 3:** Results of descriptive statistics and the covariance matrix.

Variables	Value, mean (SD)	Min	Max	VRB^a^	AL^b^	PV^c^	DoE^d^	PR^e^	IS^f^	ES^g^	SNC^h^	SI^i^
VRB	2.141 (3.334)	0	37	1	0.025	0.216^j^	−0.019	0.013	0.562^j^	0.376^j^	0.464^j^	0.490^j^
AL	188.46 (120.866)	9	894	0.025	1	−0.025	0.015	−0.071^k^	0.081^j^	0.001	0.029	0.104^j^
PV	647.985 (1918.211)	17	46,173	0.216^j^	−0.025	1	0.350^j^	−0.136^j^	0.374^j^	0.110^j^	0.026	0.302^j^
DoE	73.170 (135.115)	0	2457	−0.019	0.015	0.350^j^	1	−0.234^j^	0.113^j^	−0.052^l^	−0.031	0.164^j^
PR	.835 (.193)	0	1	0.013	−0.071^k^	−0.136^j^	−0.234^j^	1	−0.189^j^	−0.002	0.094^j^	−0.230^j^
IS	3.274 (1.467)	1	54	0.562^j^	0.081^j^	0.374^j^	0.113^j^	−0.189^j^	1	0.357^j^	0.052^l^	0.135^j^
ES	4.375 (3.991)	0	14.5	0.376^j^	0.001	0.110^j^	−0.052^l^	−0.002	0.357^j^	1	0.055^l^	0.116^j^
SNC	.536 (.610)	0	5.333	0.464^j^	0.029	0.026	−0.031	0.094^j^	0.052^l^	0.055^l^	1	0.589^j^
SI	8.75 (8.757)	1	88	0.490^j^	0.104^j^	0.302^j^	0.164^j^	−0.230^j^	0.135^j^	0.116^j^	0.589^j^	1

^a^VRB: voluntary rewarding behavior.

^b^AL: answer length.

^c^PV: page view.

^d^DoE: date of exposure.

^e^PR: provider reputation.

^f^IS: informational support.

^g^ES: emotional support.

^h^SNC: social norm compliance.

^i^SI: social interaction.

^j^*P*<.001.

^g^*P*<.01.

^j^*P*<.05.

### Data Analysis

As our dependent variable (ie, *voluntary rewarding behavior*) is count data, we used count data models for our analysis [106]. As the variance value of VRB (11.114) is greater than its mean value (2.141), the distribution of the dependent variable was overdispersed, and a negative binomial (NB) model is preferred over a Poisson model [107]. NB regression relies on a log-transformation of the conditional expectation of the dependent variable and requires an exponential transformation of the estimated coefficients for assessing and interpreting the effect sizes [108]. Following econometric modeling guidelines and based on Stata 15 [106], we tested our hypotheses by using the *nbreg* model with the following equation:

*Log(λ(VRB_i_*))=β_0_ + β_1_*ArticleLength_i_* + β_2_*PageView_i_* + β_3_*DoE_i_* + β_4_*ProviderReputation_i_* + β_5_*InformationSupport_i_* + β_6_*EmotionalSupport_i_* + β_7_*SocialNormComplaince_i_* + β_8_*SocialInteraction_i_* + *ε_i_*

Where *λ_i_*=exp(*x_i_ + offset_i_*), represents a vector of parameters for the model predictors, *x*_i_ represents the i^th^ predictor, and *ε_i_* represents the i^th^ error term.

## Results

### Hypothesis Test

We ran the NB model with the volume of *voluntary rewarding behaviors* as the dependent variable. The overall results indicated a good fit with a highly significant log likelihood ratio (*P*<.001 for Wald^2^; see Table 4).

**Table 4:** Results of the negative binomial model (N=2148).

Indices^a,b,c^	Results			
	Coefficient	SE	Z test	*P* value>Z test value
Constant	0.367^d^	0.021	17.180	<.001
Response length	−0.033^e^	0.019	−1.780	.07
Page view	0.072^d^	0.017	4.220	<.001
Date of exposure	−0.050^f^	0.022	−2.250	.02
Provider reputation	0.135^d^	0.023	5.960	<.001
Informational support	0.168^d^	0.020	8.540	<.001
Emotional support	0.463^d^	0.023	20.490	<.001
Social norm compliance	0.510^d^	0.018	28.150	<.001
Social interaction	0.281^d^	0.021	13.230	<.001
Social interaction×informational support	0.032^f^	0.013	2.410	.02
Social interaction×emotional support	−0.086^d^	0.006	−13.600	<.001
Social interaction×social norm compliance	0.014^g^	0.016	0.880	.38

^a^Log likelihood=−3130.778.

^b^Likelihood ratio^2^_11_=2178.5 (*P* value<.001).

^c^Pseudo R^2^=0.258.

^d^*P*<.001.

^e^*P*<.1.

^f^*P*<0.05

^g^Nonsignificant.

### Findings

As shown in Table 4, most hypotheses were supported (our tests are 2-tailed tests and the degree of freedom is 11). The four direct effects were significant. Informational support (β=.168; *t*_11_=8.540), emotional support (β=.463; *t*_11_=20.490), social norm compliance (β=.510; *t*_11_=28.150), and social interaction (β=.281; *t*_11_=13.230) positively influenced consumers’ VRBs in OHCs. H1, H2, H3, and H4 were supported. The moderating effects of social interaction on informational support (β=.032; *t*_11_=2.410) and emotional support (β=−.086; *t*_11_=13.600) were significant. H5 and H6 were supported. The moderating effect of social interaction on social norm compliance (β=.014; *t*_11_=0.880) was insignificant. H7 was unsupported.

Although we proposed that social interaction negatively moderates the effect of social norm compliance on consumers’ VRBs, our results did not support this hypothesis. This may be because although CEST indicates such a negative moderating effect [70], other literature suggest that social interaction can provide consumers an opportunity to observe what others do [39,91], ie, the more frequently health service providers and consumers interact, the more consumers feel social pressure from others and the expectation to fit within social norms. This may be likely to enhance the effects of social norm compliance to some extent and that is why we did not observe a significant relationship empirically.

## Discussion

On the basis of prior related studies and grounding our research in CEST, this study has identified two health service content–related factors and two interpersonal factors and explored how these factors influence consumers’ VRBs toward free health service contributors in OHCs. Our empirical findings have demonstrated that informational support, emotional support, social norm compliance, and social interaction positively influence consumers to voluntarily reward free health service contributors. In addition, social interaction enhances the effect of informational support but weakens the effect of emotional support on consumers’ VRBs toward free health service contributors in OHCs.

### Theoretical Contribution

This paper makes two theoretical contributions. First, we contribute to the literature on knowledge sharing in OHCs. As noncommercial web-based SE platforms are becoming increasingly popular, scholars have begun to examine health care professionals’ or consumers’ health knowledge–sharing behaviors [6,9,11,22,32]. However, few studies have explored the factors influencing consumers’ VRBs, which is an effective way of promoting the sustainable provision of health services in OHCs. This study has addressed this gap. On the basis of prior studies, we identified two health service content–related factors (ie, informational support and emotional support) and two interpersonal factors (ie, social norm compliance and social interaction). On the basis of CEST, we verified that informational support, emotional support, and social norm compliance positively influence consumers’ VRBs, and social interaction, as an external factor, also positively influences consumers’ VRBs. Social interaction enhances the effect of informational support but weakens the effect of emotional support. Given that the VRBs toward free web-based health service contributors is so new that it has not been studied well, the abovementioned findings contribute to the research on knowledge sharing by identifying and explaining how different factors motivate consumers to voluntarily reward free health services in OHCs.

Second, our research is based on CEST and also contributes to CEST. Specifically, CEST mentioned that the extent to which individuals behave primarily according to one of the systems varies based on situations or the person himself or herself [70,95], but it did not specifically study which factor can affect such changes. Some later studies have verified the abovementioned proposition in different situations and found that external factors (eg, attraction effect and constructive thinking) do change the effects of experiential and rational systems [81,82]. This study has verified the abovementioned proposition in an OHC context. We found that social interaction together with emotional support negatively influences consumers’ VRBs, but together with informational support, it positively influences consumers’ VRBs. This finding extends the literature on CEST by verifying the moderating roles of a new external factor (social interaction) in a new context (OHCs).

### Practical Implication

This paper has identified and verified the effects of four main variables on consumers’ VRBs on free health services in OHCs. We contributed to noncommercial web-based SE platforms by providing these platform operators strategies on how to motivate consumers to voluntarily reward free service contributors.

First, platform operators could optimize their platform feature design. They can optimize the platform communication features and encourage service providers and consumers to interact with each other. In addition, they can design and implement new rewarding systems. For example, they can display the rewarding messages such as “consumer XX just rewarded provider YY some money.” These rewarding messages might cause more consumers to comply with others and choose to reward free service contributors.

Second, platform operators should encourage service providers to contribute professional knowledge and generate high-quality services. They can invite more professionals or experts to use their platforms. They can help enthusiastic consumers to improve professional capabilities. The engagement of professionals and enthusiastic consumers can guarantee the quality of services on noncommercial SE platforms and can in turn attract more consumers to use their platforms and reward free service contributors.

### Limitations for Future Studies

We address two potential limitations. First, we did not test the effects of consumers’ sociodemographic variables and consumer characteristics. As the dataset was crawled in a public community, we could not obtain consumers’ sociodemographic information and their characteristics. In addition, we measured all variables with the objective data, namely an indirect measurement approach. Second, different from prior studies that use the actual volume of money as dependent variables, we used the number of times a thread is being rewarded as the dependent variables. We are not sure whether these points undermine our conclusions or not. We appeal that more studies be conducted through the econometric modeling approach and also suggest a mixed method approach of combining objective data and subjective data in future studies.

## Abbreviations

CEST: cognitive-experiential self-theory
ICT: information and communication technology
NB: negative binomial
OHC: online health community
SE: sharing economy
VRB: voluntary rewarding behavior
